# Label free, quantitative single-cell fate tracking of time-lapse movies

**DOI:** 10.1016/j.mex.2019.10.014

**Published:** 2019-10-18

**Authors:** C. Elizabeth Caldon, Andrew Burgess

**Affiliations:** a The Kinghorn Cancer Centre, Garvan Institute of Medical Research, Darlinghurst, NSW, 2010, Australia; b St. Vincent’s Clinical School, Faculty of Medicine, UNSW Sydney, NSW, 2052, Australia; c ANZAC Research Institute, Sydney, NSW, 2139, Australia; d Faculty of Medicine and Health, Concord Clinical School, University of Sydney, Sydney, NSW, Australia

**Keywords:** Live cell imaging, Cell cycle, Mitosis, Interphase, Microscopy, Cell death, Anaphase, NEBD, Brightfield

## Abstract

Historically, the ability to perform multi-day time-lapse imaging of adherent cells required expensive and specialized microscopy equipment. As byproduct of this cost, many labs would synchronize cells using inhibitors such as hydroxyurea and thymidine, and or use fluorescent biosensors to minimize time required on the microscope. These methods introduce significant artefacts including phototoxicity, increased DNA replication stress and mitotic defects, thereby limiting the ability to characterize various cell cycle phenotypes. However, increased access to low cost live cell microscopes has removed many of the economic barriers thereby allowing multi-day imaging on asynchronous cells on a regular basis. Here we describe our protocol for manually tracking individual cell fates across multiple generations of random daughter cells using only low toxicity brightfield based imaging. Importantly, our pipeline relies on the free open-source software ImageJ/Fiji and an easy to use Microsoft Excel spreadsheet. Furthermore, annotated files can be saved to allow later recall of any individual cell. In summary, our method provides quantitative data on interphase and mitotic transit time, points of cell cycle arrest and critically, the ability to link these events with cell fate.

## Graphical abstract

**Figure:**
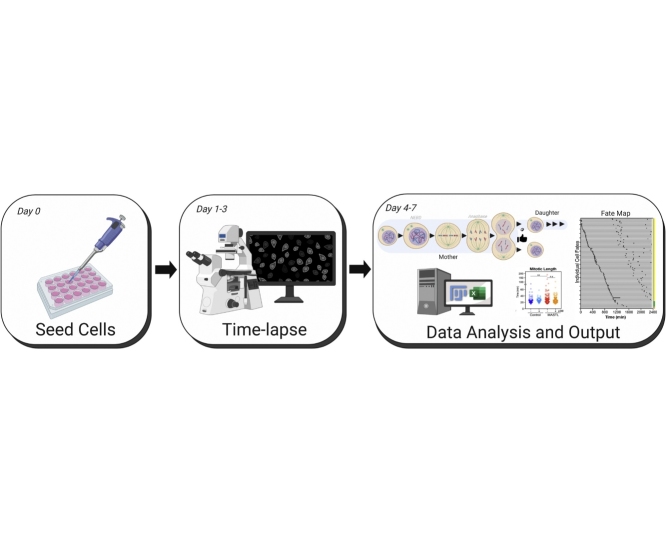

**Specification Table**

**Table:** 

Subject Area:	*Biochemistry, Genetics and Molecular Biology*
More specific subject area:	*Cell Biology*
Protocol name:	*Label Free Live Cell Imaging Analysis*
Reagents/tools:	*Fiji/ImageJ, wide-field microscope with brightfield, DIC or phase contrast abilities, Microsoft Excel or other spreadsheet software.*
Experimental design:	*Adherent cancer cells were seeded on a multi well plate and placed on a live cell microscope. Cells were imaged using brightfield illumination every 5*–*10 min for up to 3 days and maintained at 37C with 5% CO_2_ for the duration using an onstage incubator.*
Trial registration:	n/a
Ethics:	n/a

**Value of the Protocol**

**Table:** 

• Enables quantitation of label free cells using time-lapse data • Produces wealth of information on individual cell fates, enabling greater insight into biological responses.

## Description of protocol

### Introduction

This protocol details the extraction of quantitative information including cell cycle length (interphase, mitosis), fate of mother-daughter cells, mitotic phenotypes (e.g. multipolar, cytokinesis failure) and exact point of death (before, during or after mitosis) from low toxicity, brightfield time-lapse microscopy data. Please note, in order to simplify the analysis pipeline and data visualization, we chose to limit this protocol to a single random daughter cell. Similarly, detailed analysis of specific interphase stages (G1, S or G2) along with subtle mitotic defects, such as ultra-fine chromosome bridges, still require the use of secondary markers or fluorescent biosensors (e.g. FUCCI, PCNA, H2B). Consequently, this method is best used to identify substantial phenotypic differences between different treatment conditions. It is also useful as an initial screening tool to extract and identify potential mechanism of action that can then be confirmed with more detailed biosensors. This has the significant advantages of not needing to spend precious time and money introducing unnecessary biosensors into various cell lines and also allows analysis of difficult to transfect cells.

### Experimental design

**Fig. 1:**
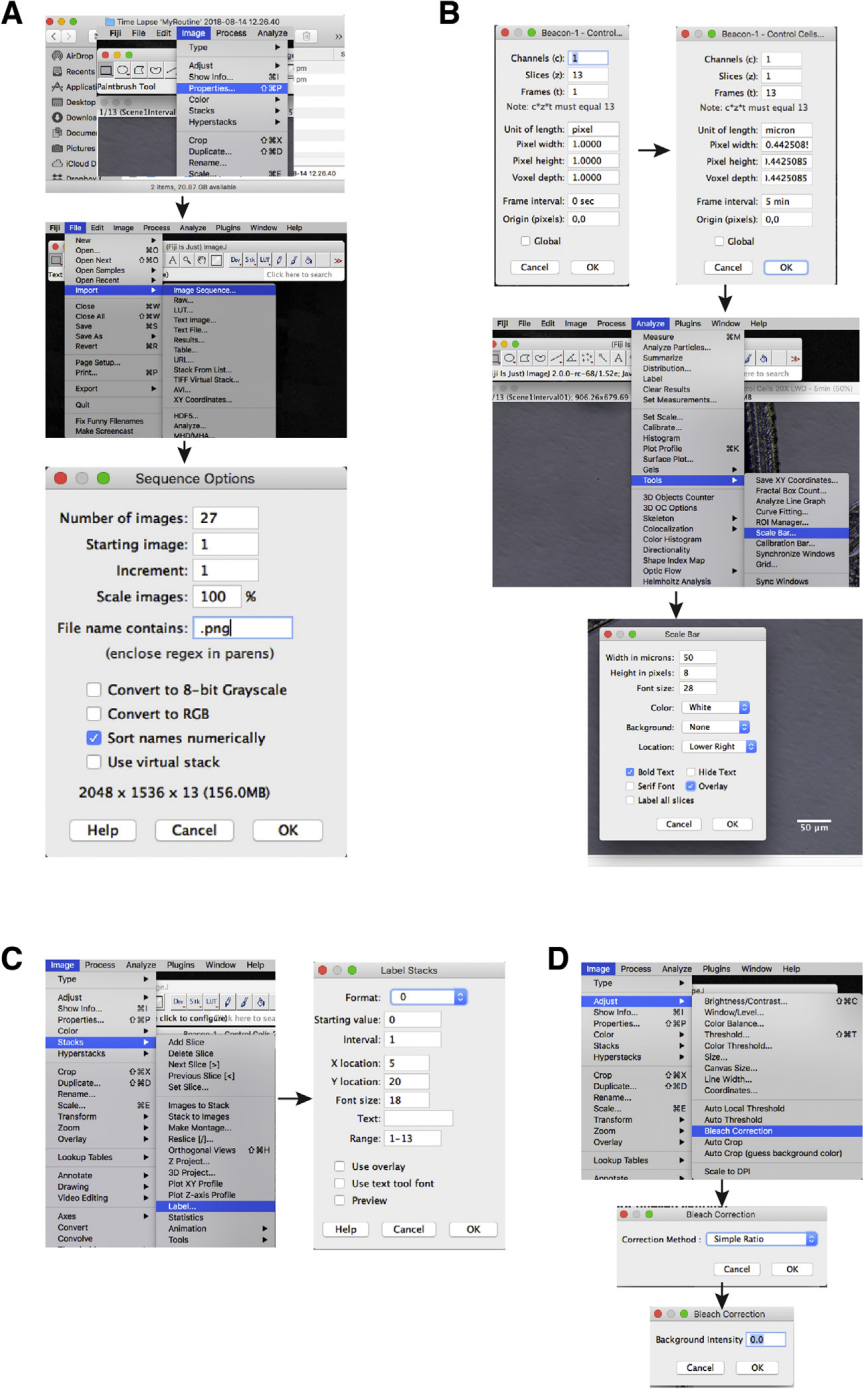
Steps to process your time-lapse movies in Fiji. (A) Importing data. (B) Scaling the data. (C) Inserting a time counter. (D) Adjusting for uneven lighting.
C **Basic Movie Editing**
  1) To check image scale information, go to: Image > Properties…
  2) If unit of length is in pixels, then your image is not scaled correctly (Fig. 1B). You will need to determine the pixel length in microns (μm) which will be dependent on the microscope, lens and camera. This can be worked out empirically using a calibration slide. Once you know this value enter it in the width section, the height and depth should auto populate and change the unit of length to ‘micron’.
  3) (Optional) In Image > Properties…input the time frame (t) interval.
  4) To insert a scale bar, go to: Analyze > Tools > Scale Bar… Change the values to what you want and select overlay to ensure scale bar appears on all frames (Fig. 1B). If you want to place a scale bar in a specific place, first draw a line at the point your scale bar is to be inserted, and then go to the Analyze > Tools > Scale Bar…menu.
  5) To insert a time counter, go to: Image > Stacks > Label… choose the format, interval length and click ok (Fig. 1C).
  6) If you have uneven lighting, you can easily correct this using Bleach Correction: Image > Adjust > Bleach Correction (Fig. 1D). Select Simple Ratio from the drop-down menu and select ok. A box will appear where you can adjust the background intensity. For most movies, leaving the background intensity at 0.0 works well, however if it doesn’t try increasing the value to 1 or more until you see improvements.
  7) Save the movie as a. tiff. You can also save as an. avi for use in presentations and publications. *(Tip) If you prefer to analyze in Adobe Photoshop CC rather than Fiji, save. avi files at 30 fps. Newer versions of Photoshop may not read. avi files correctly, in these cases convert the files to. mp4 (H.246) format prior to importing into Photoshop. This has the advantage of much smaller file size for long-term storage of analysis data.*
D **Identifying Cell Types**
  1) Interphase and mitotic cells can be easily identified in most adherent cells due to the clear phenotypic changes. We score mitotic length as the time between nuclear envelope breakdown (NEBD) to anaphase, and randomly follow daughter cells to generate multigenerational fate maps (Fig. 2A).

A **Setup of Time-lapse**:
  Any adherent cell line can be used for this method. We regularly use and have published this method with human cancer lines including A549, H358, HeLa, Hs578 T, MD-MB-231, MCF-7 and MCF10A [[1], [2], [3]].
  1) Cells are seeded at approximately 30% confluency the day prior to imaging onto an appropriate multi-well plate*. (Tip) We regularly use standard plastic 6, 12 and 24 well plates coupled with a long working distance 10X or 20X microscope lens. If magnification above 20X is needed, or there is no LWD lens available, the use of thin optical plastic or glass bottom dishes is usually required.*
  2) On day of imaging cells should be no more than 40–50% confluent. Place plate on to the microscope and select 2–3 random fields per well/condition. *(Tip) We have used this method on various inverted microscopes including a Zeiss Axiovert 200 M, EVOS FL Auto 2, Leica DMi8 and an IncuCyte, but any inverted live cell microscope will work.*
  3) Adjust brightfield, DIC or phase settings to maximize image quality while minimizing exposure time. Note excessive light (especially fluorescent) exposure can be toxic to cells and will cause a mitotic delay [4].
  4) (Optional) set up fluorescent channels for any biomarkers or dyes that you might be using. For example, propidium iodide (1 μg/ml) can be added to the media as a low-cost fluorescent marker of cell death.
  5) Depending on your needs, set image acquisition time from between 5 to 20 min. If highly accurate mitotic length analysis is required, intervals of 5 min or less is preferred, as a normal mitosis is approximately 20–40 min in length, and hence longer time frames will often miss key events. If imaging at intervals <10 min it is essential that you minimize exposure time and lamp intensity, especially on fluorescent channels, to avoid light toxicity.
  6) Upon completion of imaging, save and export images as either. jpg,. png or. tiff. Note, some microscopes have their own image format, which ImageJ/Fiji can often import. The advantage of these files and. tiff is that scale information is usually embedded in the image meta-data. *(Tip) Make sure you have written down what each or position/beacon is, we suggest renaming the folders so that it is easier to remember.*
B **Importing data into ImageJ/Fiji**
  1) Download and install Fiji [5] from https://fiji.sc (note, Fiji is just ImageJ, with many popular plug-ins pre-installed).
  2) Import your time-lapse data using: File > Import > Image Sequence (Fig. 1A). If you have multiple file types in the folder you can limit importing to only files of interest by including a qualifier such as. png (or. tiff) in file name contains box. *(Tip) if you don’t have a lot of installed memory, select ‘use virtual stack’, convert to greyscale, and/or skip frames if you don’t need all of the timepoints*, *to reduce the file size.*

**Fig. 2:**
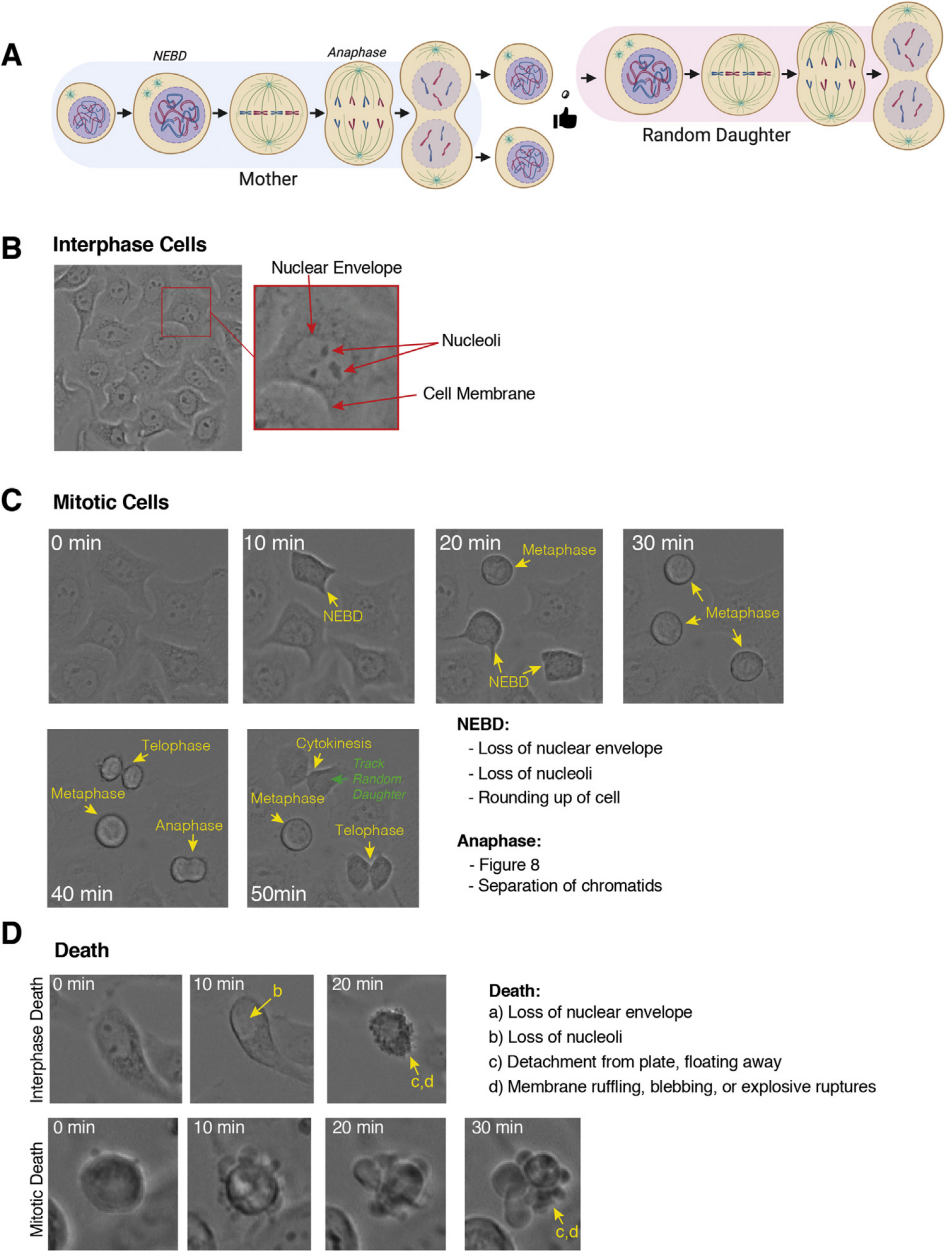
Identifying cells on your time-lapse movies. (A) Schematic of the cell cycle across two generations. (B) Identifying interphase cells. (C) Characterising the steps in mitosis. (D) Recognising cell death.
  2) NEBD is characterised by the one or more of the following: loss of nuclear envelope, loss of nucleoli and/or rounding up of the cell (Fig. 2B, C). Anaphase is characterised by one or more of the following: separation of chromatids and membrane invagination creating a ‘figure 8’ appearance (Fig. 2C). *(Tip) if >5 min intervals are used specific mitotic phases are often missed. In this case, score the first frame after NEBD or anaphase has occurred, but be aware the timing is less accurate.*
  3) (Optional) Cytokinesis can also be scored, although this is more difficult to score as the breakage of the fine cytoplasmic bridge between daughter cells is often difficult to observe (Fig. 2C).
  4) Death is commonly observed and characterized by the following: Loss of nuclear envelope, loss of nucleoli, detachment from plate, floating away in combination with extensive membrane ruffling, blebbing, or explosive ruptures (Fig. 2D). *(Tip) Addition of propidium iodide can facilitate identification of death, by highlighting cells that have lost membrane integrity, while fluorescent caspase markers can be used to specifically identify apoptosis.*
E **Quantifying Movies and Single Cell Fate Mapping using Fiji/ImageJ and Excel**
  1) Open your saved. tiff file from step C-7 above.
  2) Enable the multi-point tool by right clicking on the point tool in the default startup Fiji tools.
  3) Click on the first cell of interest, and it will be marked with a #1. Follow it through the time-course until you encounter NEBD or death.
  4) Note the time in the excel spreadsheet for NEBD, Anaphase, or when the cell died (see section F below).
  5) Mark down any mitotic phenotypes observed (e.g. multipolar, cytokinesis failure).
  6) For daughter cell analysis, randomly pick one daughter and follow it.
  7) Repeat 4–5 until you reach the end of the movie.
  8) Return to frame one and select another cell (#2) using the multi-point tool.
  9) Repeat the process until you have ideally counted 50–100 cells.
  10) Save the. tiff file. This will keep the counted cell labels allowing you to return at any time to find a cell of interest or to continue counting more cells.
F **Using Excel to track data and generate Cell Fate Maps**
  1) Open the excel movie template (Supplementary File S1).
  2) (Optional) Add-in additional grand-daughter columns as needed, for example if you are performing long 3–5 day movies.
  3) Delete the demo data (Column B–K only) and then duplicate both the Condition #1 and Fate Map #1 sheets depending on the number of conditions you wish to analyze.
  4) Follow Cell #1 through the movie noting in Row 3 the time for NEBD, anaphase, death and any phenotypes identified in the respective columns. (*Tip) Update the running total list at the end with any additional phenotypes you observe.*
  5) Continue following a random daughter/grand-daughter cell and note these in their Row 3 columns*. Note a limitation of this analysis pipeline is that the fate of only a single random daughter cell is tracked.*
  6) Repeat for Cell #2 onwards until you have counted sufficient cells, ideally 50–100 per condition. *(Tip) We find that most users can normally track around 25–50 cells/hour.*
  7) Once all cells have been counted, the data in columns M-Q needs to be edited.
  8) Update the formula in Q3 to match the total movie length and apply formula from Q3 down to the end of the values, depending on how many cells you have counted (Fig. 3A).

**Fig. 3:**
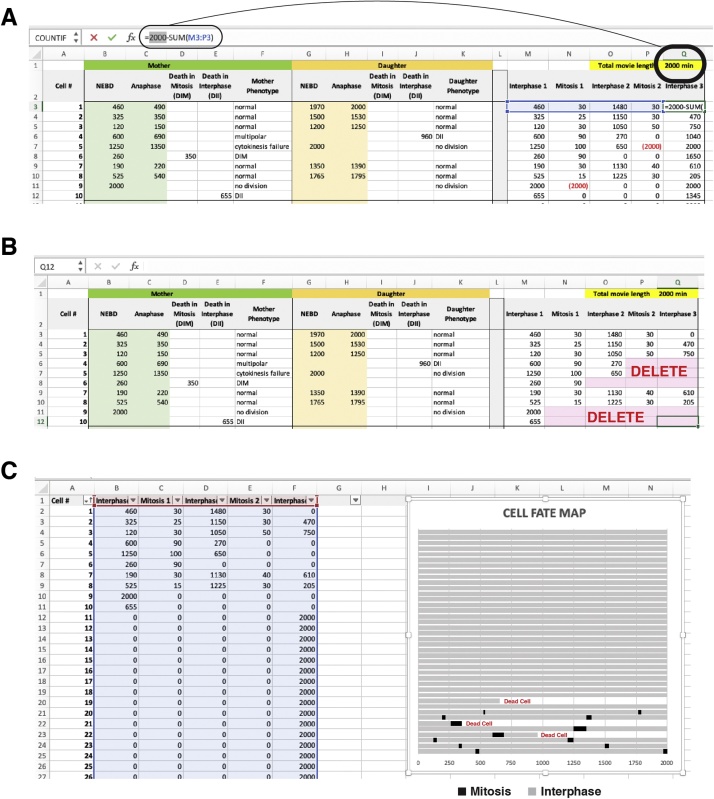
Creating single cell fate maps in Excel. (A) Adjusting for the total movie length. (B) Adjusting the data for death events. (C) Visualising the fate map.
  9) DELETE negative (RED) values along with any number to the right of the negative number. In addition, delete any values to the right of any death event (Fig. 3B).
  10) Select the Fate Map #1 tab to visualize your preformatted fate-map (Fig. 3C). *(Tip) Re-sort the columns (ascending/descending) to group cells according to phenotypes, and this will make different phenotypes more apparent.*
  11) To calculate the mean mitotic length across all generations, combine values from mitosis 1 and 2 columns (Column C and E) and generate dot-plots. Calculate statistical significance using statistical software (e.g. Graphpad Prism (www.graphpad.com), PlotsofData [6]). Similarly, total cell cycle length between mother and daughter cells can be calculated by plotting all Interphase #2 values. *(Tip) You can generate multiple graphs of data for whatever best suits your biological question, for example you could keep mother-daughter cell data separate to identify any cell cycle defects caused during the 1^st^cell division.*
